# The Effect of Two Different Wavelengths of Diode Laser on the Shear Bond Strength of Composite to Dental Enamel after Bleaching Process: An In Vitro Study

**DOI:** 10.3390/bioengineering11060559

**Published:** 2024-06-01

**Authors:** Reza Pourmahmoudian, Luca Solimei, Stefano Benedicenti, Sogol Saberi, Sima Shahabi

**Affiliations:** 1 School of Dentistry, Tehran University of Medical Sciences, Tehran 1416634793, Iran; 2 DISC—Department of Surgical Sciences and Integrated Diagnostics, University of Genoa, 16126 Genoa, Italy; lucasolimei@hotmail.it (L.S.); benedicenti@unige.it (S.B.); 3 Laser Research Center of Dentistry, Dentistry Research Institute, Tehran University of Medical Sciences, Tehran 1416634793, Iran; 4 Department of Dental Biomaterials, Tehran University of Medical Sciences, Tehran 1416634793, Iran

**Keywords:** diode laser, tooth bleaching, dental enamel, composite resin

## Abstract

Introduction: In recent years, tooth whitening has become one of the most popular ways of achieving the original tooth color. The effect of whitening gel can be improved through heat, light or laser. The bond strength between the enamel and the composite can be reduced through bleaching and laser radiation. The purpose of this study is to assess the shear bond strength of resin composite to enamel after a bleaching process using hydrogen peroxide, with and without a laser (970 nm and 445 nm lasers). Method: This study used 51 extracted anterior teeth without caries that were divided into three groups. A 40% hydrogen peroxide gel was used on the enamel of all teeth. The control group received bleaching without a laser. Both the second and third treatment groups received bleaching with a laser, one with 970 nm and the other with 445 nm. After the bleaching process, all groups had etching, bonding and curing of the composite performed. Lastly, the shear bond strength between the enamel and the composite was measured and the failure modes were recorded. The data were compared using a one-way ANOVA test. Results: The mean shear bond strength between the enamel and the composite in the 445 nm group three (445 nanometer) was significantly lower than the other groups (*p* < 0.05). There was no significant difference between the control and the 970 nm groups (*p* = 0.2). Conclusion: According to the laser wavelengths and parameters that were used in this study and the results of this study, office bleaching with a 445 nm laser weakened the shear bond strength between the enamel and the composite.

## 1. Introduction

Tooth discoloration is divided into internal and external discoloration. Internal tooth discoloration factors include trauma, blood-related illnesses, tetracycline intake, dental caries and restorative materials. External tooth discoloration occurs due to either the consumption of pigmented foods and drinks, mouthwash and/or poor oral hygiene [1,2,3,4]. Internal tooth bleaching is performed for internal discoloration and external tooth bleaching is performed for external discoloration. For internal tooth bleaching, carbamide peroxide or hydrogen peroxide are used in root-canal-filled teeth. External bleaching has become very popular in recent years, and it is performed in two ways; in office and at home. The whitening agent in the bleaching process can be hydrogen peroxide, carbamide peroxide and sodium percarbonate. Hydrogen peroxide gel is used for office bleaching, as it works quickly. Carbamide peroxide gel is used for home bleaching, and it is similar to hydrogen peroxide, but it takes more time, and it is more stable. The other whitening option, sodium percarbonate, is less sensitive for teeth and is combined with hydrogen peroxide [4,5].

Hydrogen peroxide gel is the most popular whitening agent. Hydrogen peroxide is an unstable material that turns into water and oxygen free radicals over time. Oxygen free radicals are combined with pigment molecules to break down pigments making them smaller and lighter, and, therefore, easier to remove. We can accelerate this process by adding an energy source like radiation or heat to the hydrogen peroxide. Radiation, heat and time accelerate the decomposition of hydrogen peroxide and the production of oxygen free radicals [4,5].

Patients who desire beautiful white teeth, especially anterior ones, choose external bleaching. However, if there is a tooth with caries, it needs to be restored with composite resin for aesthetic reasons. Bleaching makes the tooth color whiter and brighter and reveals the original color of the tooth. We perform the bleaching first for patients who have caries, and in order to pick the right composite resin color, we need the original tooth color without any pigments. We have to choose the composite color based on the natural tooth color so that the results are whiter. If we were to perform the composite restoration before bleaching, the tooth would be whiter than the composite and vice versa. The restoration and tooth color should be the same and consistent, therefore, we have to perform the bleaching first.

Using a high percentage of hydrogen peroxide roughens the enamel surface [6,7] and causes enamel hydroxyapatite crystals to change, thus, weakening the bond between the enamel and the composite [7,8,9,10,11,12] depending on the time spent, type of radiation and method. The more intense the bleaching, the more structural changes there are to the enamel, thus, making the bond strength between the enamel and the composite weaker. Research shows that bleaching with radiation is more effective than bleaching without any radiation [6,13,14]. Laser radiation weakens the enamel to composite bond strength because of the structural changes to the enamel [8,10,14,15].

Some of the different radiation devices used in bleaching emit a single specific wavelength called a laser [8,15], while others emit a range of wavelengths, like the QTH (quartz–tungsten–halogen) light-curing device, which emits a 380 to 520 nm light [8]. Nowadays, there are different laser devices with different wavelengths. One of them is the diode laser device, which is widely used and has different wavelengths. In recent years, studies have been conducted on the effects of diode lasers in dental treatments. However, the 445 and 970 nm diode laser wavelengths are relatively new and not many studies have been conducted on them. The shorter the laser wavelength measured with a nanometer, the higher the energy [16]. The 445 nm laser has more energy than the 970 nm laser. The 445 nm laser is a blue laser with a stronger absorption rate in pigments, melanin and hemoglobin, and, due to the shallow penetration of this laser, has fewer possible injuries in the deep layers of the tooth [17].

The purpose of this study is the in vitro comparison of the ability of two diode lasers with different wavelengths to change the shear bond strength of the composite to the enamel. Some research has been conducted on bond strength after bleaching, but it has mostly compared different types of lasers and different methods [8,10,13,14,15]. Considering that the wavelength of 445 nm is a new wavelength and there are not many studies in this field, and its comparison with the wavelength of 970 nm has not been performed in the subject of a study, and considering that both bleaching and composite treatments are increasing, our goal is to compare the 445 nm diode laser to the 970 nm diode laser based on a non-laser bleaching control group, something that has not yet been tested at these specific wavelengths in terms of bond strength. The null hypotheses to be tested were as follows: the shear bond strength of composite to enamel after bleaching in the diode laser group with a 445 nm wavelength, the diode laser group with a 970 nm wavelength and the control group without a laser, having no significant differences.

## 2. Methods

The ethical approval code of this study is IR.TUMS.BLC.1401.036. The purpose of this study was to measure the shear bond strength of enamel to composite resin after bleaching with 40% hydrogen peroxide accelerated using two diode lasers with wavelengths of 445 nm and 970 nm. Further, the two laser wavelengths were compared to each other, as well as to the non-laser bleaching control group.

This study was conducted on 51 human incisor and canine teeth that had been extracted due to periodontal problems and were without caries, restorative work or cracks. For disinfection, the samples were immersed in a 0.5% chloramine solution at room temperature (23 °C) for one week. After that, there were randomly divided into three groups (two laser groups and one non-laser control group) using Excel. Next, the roots of the samples were cut 2 mm under the CEJ (cementoenamel junction) using a diamond disk. After that, the tooth crowns were mounted horizontally on acrylic resin so that their buccal sides were visible and facing up. Lastly, the buccal surfaces were polished using sandpaper.

Samples in all groups were washed and dried. Hydrogen peroxide gel (Boost chemical activation—H_2_O_2_ 40%Ultradent—Opalescence, NC, USA) was used in all groups. In group one (control group), bleaching gel was applied to the samples for 20 min, according to the manufacturer’s instruction, without any laser radiation, followed by the rinsing of the samples. In group two, hydrogen peroxide gel was applied with a 970 nm laser radiation device (Siroblue laser, dentsplySirona, NC, USA) and, using a handpiece (multi-tip 8 mm), 2 watts of power were applied for 30 s, followed by the rinsing of the samples. In group three, hydrogen peroxide gel was applied with a 445-nanometer laser radiation device (Siroblue laser, dentsplySirona, Germany) and, using a handpiece (multi-tip 8 mm), 1.5 watts of power was applied for 20 s, followed by the rinsing of the samples [17]. In the second and third group, this process was repeated three times with an interval of one minute. The total bleaching time in the first group was 20 min, while the second group was 3 min and 30 s, and the third was 3 min.

All the samples were washed after bleaching and were immersed in artificial saliva (37 °C) for two weeks. After that, the enamel surfaces of all samples were etched using 35% phosphoric acid (Cobalt Etch) for 30 s and were then rinsed. Then, the enamel surface was dried using an air and bonding agent (GLUMA^®^ Bond5, Kulzer GmbH, Hanau, Germany), which was applied according to the manufacturer’s instruction using a micro brush for 15 s, followed by air drying for 5 s. After that, the bonding agent was cured for 20 s using a LED (light-emitting diode) light-curing device (Kerr, Orange, CA, USA). Then, A2 composite resin (Filtek Z250; 3M ESPE, St. Paul, MN, USA) was placed on the prepared enamel surface in a cylindric shape (height of 2 mm and inner diameter of 2 mm) and was cured for 40 s using a LED light-curing device (Kerr, Orange, CA, USA).

After that, the samples were immersed in distilled water (37 °C) for two weeks. Next, the shear bond strength of the enamel to the composite was measured using a universal testing machine (SANTAM, SMT-20, Tehran, Iran) at a crosshead speed of 0.5 mm/min until debonding occurred. Lastly, samples were observed using a stereomicroscope (Olympus SZ 4045 TRPT, Tokyo, Japan) at x10 magnification to evaluate the site of failure.

Considering that the data had a normal distribution using the Shapiro test, we compared the groups with the one-way ANOVA test, and the three groups had differences. We used the Tukey HSD test to compare two groups according to the equality of variance. A significance level of less than 0.05 was considered. A statistical analysis was performed with IBM SPSS 25 software; considering α = 0.05 and β = 0.2, the average standard deviation of the shear bond strength was equal to 5.5 MPa and the effect size equal to 0.45, the minimum sample volume required for each of the 3 study groups was equal to 17 samples.

## 3. Results

**Figure 1:**
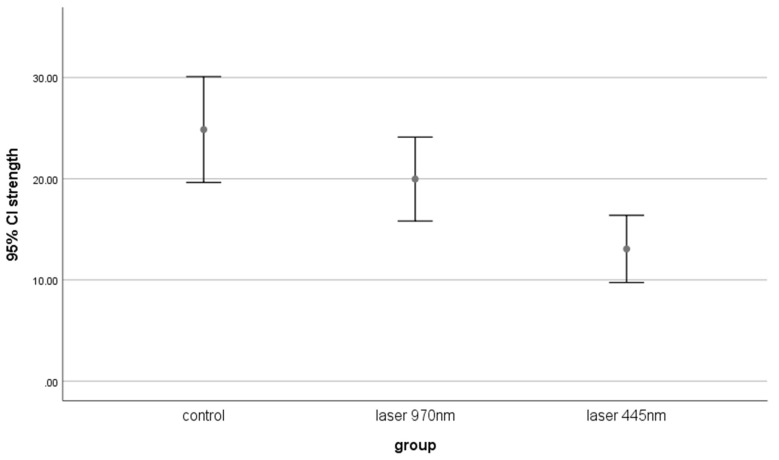
Shear bond strength of composite to enamel in all groups after bleaching (error bar and 95% confidence level).

The mean, minimum and maximum shear bond strengths (MPa (megapascal)) of the groups are shown in Table 1 and Figure 1. The control group had the highest shear bond strength between the enamel and the composite, while the 445 nm laser group had the lower. The data analysis using ANOVA showed significant differences between the groups (*p* < 0.001). The groups were compared using the Tukey HSD test.

**Figure 2:**
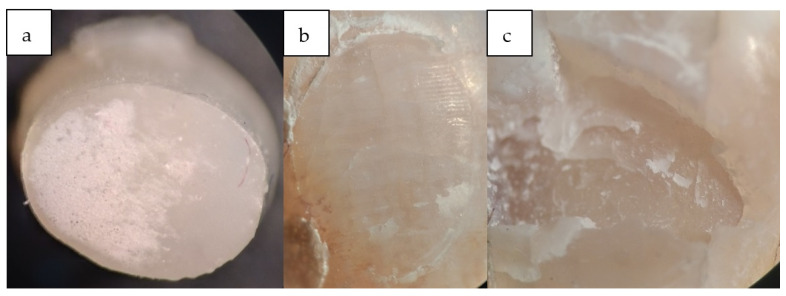
Modes of bond failure. (**a**) Composite surface in adhesive bond failure. (**b**) Enamel surface in adhesive bond failure. (**c**) Enamel surface in the failure of the cohesive bond.

There was no significant difference between the non-laser control group and the second group (970 nm laser) (*p* = 0.2). The mean shear bond strength in the control group was significantly higher than in the 445 nm group (*p* < 0.001). The mean shear bond strength in the 970 nm group was significantly higher than in the 445 nm group (*p* = 0.049). The failure mode of all groups is shown in Table 2. Examples of the failure mode are shown in Figure 2. The failure mode was mostly adhesive failure in all groups. Not using more wavelengths and different parameters was one of the limitations of this study. Also, a thermocycler was not used in this study, and the samples were not subjected to thermal stress. Heat stress could have led to different results. It is suggested to study lasers with different wavelengths and parameters in future studies.

## 4. Discussion

The shear bond strength of enamel to composite is affected by the enamel surface, as bleaching changes the surface. The loss of the prismatic structure of the enamel and the loss of calcium and changes in the structure of the enamel cause porosity and weaken the adhesive properties of the enamel to the composite [7,8,9,10,11,12]. Conventional bleaching systems require more time to be effective, and can cause more demineralization, while the reactions in laser whitening happen faster than conventional methods and, as a result, the demineralization of tooth enamel after laser whitening can be milder [18].

Our results showed that the average shear strength of the bond between enamel and composite in the 445 nm group was significantly lower than the other groups (*p* < 0.05). There was no significant difference between the control group and the 970 nm group (*p* = 0.2). This could be because, according to previous studies [19], the difference between the 445 nm and 970 nm groups could be due to the shorter wavelength of the 445 nm laser. The penetration depth of the 445 nm laser is greater than that of the 970 nm laser. Also, the 445 nm wavelength has a high absorption in the red pigment [20], and the bleaching gel used in our study was also red, so it could have a high absorption with the 445 nm wavelength. All these things caused an increased production of oxygen free radicals, which caused more unevenness in the enamel surface and decreased the strength of the bond.

In some past studies, according to the gel used and the amount of hydrogen peroxide in it or carbamide peroxide instead of hydrogen peroxide, a change in the shear bond strength was seen after laser bleaching [8], and in others it was not seen [9,10].

Unlu et al. showed that applying a bonding process two weeks after bleaching had the best result in terms of the shear bond strength of the enamel to the composite [11], which was similar to our study. Also, Cavalli et al. found that applying a bonding process three weeks after bleaching was not significantly different than the non-bleaching control group in terms of the shear bond strength of the enamel to the composite [12].

After curing the composite, the teeth were immersed in distilled water for two weeks, because the curing and polymerization of composite resin takes time. The more time that passes, the more the number of monomers in the composite decreases and the composite strength increases [21].

In the current study, usual bleaching gel with the help of a laser was used; in the study of Azarbaijani et al., two groups out of five studied groups were bleached with conventional gel with 810 and 980 nm lasers, and it was shown that groups with conventional gel showed more noticeable increases in crystallinity. This implied a better interaction of conventional bleaching gel with a diode laser compared to the interaction of a laser with the used laser-activated gel, which could justify our better results regarding the wavelength of 970 nm [22].

Alaghehmand et al. prepared class V cavity samples with bonded and cured composite, and then performed the bleaching process. They used 25% hydrogen peroxide gel. They found no significant difference between the non-laser bleaching group and the group bleaching with a 815 nm diode laser in terms of the shear bond strength of the enamel to the composite [13], which was similar to our result of the 970 nm laser.

Ergin et al. used a 940 nm laser and 35% hydrogen peroxide for bleaching and found that the shear bond strength of the enamel to the composite was reduced significantly post-bleaching compared to pre-bleaching. They did not have a control group [14].

Ceyda et al. showed that the non-laser bleaching control group had the highest shear bond strength of the enamel to the composite, while the bleaching group with a 940 nm diode laser had a significantly lower shear bonding strength of the enamel to the composite compared to the control group [15]. The difference to our results might have been caused by different methodologies, such as differences in the power and wavelengths.

In general, some studies have shown that a light-emitting diode laser in bleaching causes a significant decrease in the shear bond strength compared to the control group, while some studies showed no significant difference between the laser groups and the control group. In conclusion, the results of each study depended on laser’s wavelengths, methods and parameters used.

Past studies have compared different types of lasers in terms of the shear bond strength [8,10,13,14,15], but comparing different wavelengths of one type of laser in bleaching was not conducted yet. Our study performed this comparison with a 445 nm and a 970 nm diode laser.

Failures in all groups were mostly adhesive, which was similar to past studies [8,10,13].

One of the strengths of this study was that it was the first to evaluate the effect of wavelength on adhesion (bond strength), so the limited study size was appropriate. Also, having a control group was a strength, but the study sample size could have been higher. It is better to consider the same bleaching time for all groups in future studies and use different bleaching gels and compare them.

## 5. Conclusions

According to the limitations and laser wavelengths and parameters used in this study, it could be concluded that bleaching with a 445 nm diode laser reduced the shear bond strength between the enamel and the composite more than the control and 970 nm groups.

## Figures and Tables

**Table 1:** Shear bond strength of composite to enamel in all groups after bleaching.

Groups (17 Teeth Each)	Mean (MPa)	SD (MPa)	Min (MPa)	Max (MPa)
One (bleaching with no laser)	24.9	10.2	11.7	49.8
Two (bleaching with 970 nm laser)	20.0	8.1	5.7	35.0
Three (bleaching with 445 nm laser)	13.06	6.5	3.4	23.3

*p*-value different than no laser (*p* < 0.001) and 970 nm laser (*p* = 0.04).

**Table 2:** Failure modes of composite to enamel in all groups after bleaching.

Groups (17 Teeth Each)	Adhesive Failure (%)	Cohesive Failure (%)	Mixed Failure (%)
Control (bleaching with no laser)	9 (53%)	5 (29%)	3 (18%)
970 nm laser (bleaching with 970 nm laser)	12 (70%)	2 (12%)	3 (18%)
445 nm laser (bleaching with 445 nm laser)	10 (59%)	3 (18%)	4 (23%)

## Data Availability

The data presented in this study are available on request from the corresponding author.
